# Finafloxacin Is an Effective Treatment for Inhalational Tularemia and Plague in Mouse Models of Infection

**DOI:** 10.1128/AAC.02294-20

**Published:** 2021-05-18

**Authors:** Kay B. Barnes, Mark I. Richards, Thomas R. Laws, Alejandro Núñez, Joanne E. Thwaite, Christine Bentley, Sarah V. Harding

**Affiliations:** a Defence Science and Technology Laboratory, Porton Down, Salisbury, United Kingdom; b Animal and Plant Health Agency, Surrey, United Kingdom; c MerLion Pharmaceuticals, Berlin, Germany

**Keywords:** finafloxacin, plague, tularemia

## Abstract

Infection with aerosolized Francisella tularensis or Yersinia pestis can lead to lethal disease in humans if treatment is not initiated promptly. Finafloxacin is a novel fluoroquinolone which has demonstrated broad-spectrum activity against a range of bacterial species *in vitro, in vivo*, and in humans, activity which is superior in acidic, infection-relevant conditions. Human-equivalent doses of finafloxacin or ciprofloxacin were delivered at 24 h (representing prophylaxis) or at 72 or 38 h (representing treatment) postchallenge with F. tularensis or Y. pestis, respectively, in BALB/c mouse models. In addition, a short course of therapy (3 days) was compared to a longer course (7 days). Both therapies provided a high level of protection against both infections when administered at 24 h postchallenge, irrespective of the length of the dosing regimen; however, differences were observed when therapy was delayed. A benefit was demonstrated with finafloxacin compared to ciprofloxacin in both models when therapy was delivered later in the infection. These studies suggest that finafloxacin is an effective alternative therapeutic for the prophylaxis and treatment of inhalational infections with F. tularensis or Y. pestis.

## INTRODUCTION

Francisella tularensis and Yersinia pestis are two bacterial species of biodefense interest, classified by the Centers for Disease Control and Prevention (CDC) as category A biological agents. As such, they pose the greatest risk to public health and security and have both been used in previous conflicts (1, 2). They are also both facultative intracellular pathogens that are able to invade, survive, and replicate within many cell types (3, 4). Francisella tularensis is the causative agent of the disease tularemia, the virulent subspecies *tularensis* (type A) causing the most severe infections when transmitted by the inhalational route (5). Although the number of reported cases of tularemia is low (100 to 200 cases reported annually in the United States), F. tularensis is considered a significant biological threat due to its low infectious dose and high mortality (6).

Yersinia pestis is the causative agent of plague, transmitted by fleas to rodents and subsequently to people (bubonic plague) or via an aerosol (pneumonic plague) (7). Today, approximately 90% of the cases of plague are reported in Africa, including 2,414 cases (1,878 pneumonic cases) in 2017 in Madagascar (8, 9). It is estimated to have caused 200 million deaths throughout history, and in 1995, the first multidrug-resistant strain of Y. pestis, resistant to 8 antimicrobials, was reported (10, 11). The evaluation of novel therapies is warranted to ensure we have effective treatment in the future.

For both bacterial species, following transmission as an aerosol to the lungs, infection is established, and disease progresses rapidly (7). If left untreated, disease is lethal (mortality rates of up to 60% and 70% for F. tularensis and Y. pestis, respectively); therefore, early administration of antibiotics is very important (12–14). Delayed or ineffective choices of treatment can result in rapid deterioration or a relapse of infection (13, 15–19).

The currently recommended treatment for severe cases of tularemia is streptomycin or gentamicin, with the fluoroquinolones or a tetracycline recommended for mild or moderate disease (20). Streptomycin, gentamicin, and ciprofloxacin are typically administered for 10 days or doxycycline for 14 days (1, 21). Traditionally, treatment for plague is also streptomycin or gentamicin, although to be effective, they require initiation to be within 24 h of the onset of symptoms (22). Ciprofloxacin and levofloxacin have also been approved by the FDA under the Animal Rule, with doxycycline and chloramphenicol as alternatives (1). Five human cases of plague (including one pneumonic case) were successfully treated with orally delivered ciprofloxacin in a study in Uganda from 2011 to 2014, supporting its utility for treating human cases (23). Ciprofloxacin or doxycycline would be used as postexposure prophylaxis for 14 days (F. tularensis) or 7 days (Y. pestis) if a large population required treatment (1, 21, 24).

The fluoroquinolones are attractive to treat diseases, including tularemia and plague, as they are well absorbed and widely distributed and can accumulate intracellularly (25). Finafloxacin is a novel “fourth-generation” (iteration of fluroquinolones over the years to improve penetration, spectrum of activity, etc.) fluoroquinolone that has shown to have *in vitro* activity against a range of Gram-negative and Gram-positive organisms, including Acinetobacter baumannii and Staphylococcus aureus, and efficacy against infections with these organisms *in vivo* (26, 27). Finafloxacin has been shown to have superior activity (compared to other fluoroquinolones, including ciprofloxacin) in low-pH, infection-relevant conditions and similar activity to comparator antibiotics at neutral pH, believed to be due to its ability to accumulate rapidly within cells and its low rate of efflux (28, 29). It is available as three different formulations that can be administered by the oral and intravenous (i.v.) routes and topically. The topical suspension of finafloxacin has been approved in the United States and Canada to treat otitis externa (30). A 5-day single daily dose of finafloxacin (delivered both i.v. and orally) resulted in an improved clinical outcome over a 10-day course of ciprofloxacin in a phase 2 trial of complicated urinary tract infections (UTIs) and/or pyelonephritis, and it demonstrated the ability to treat fluoroquinolone-resistant uropathogens (31, 32). In addition, *in vitro* activity has been demonstrated against all of the bacterial agents of biodefence interest, with efficacy demonstrated against inhalational infection with Burkholderia pseudomallei and intranasal infection with F. tularensis *in vivo* (33–35).

This work details an investigation into the utility of finafloxacin as an alternative prophylaxis (administered before the development of clinical signs of disease) or as treatment (upon development of clinical signs), following inhalational infections with F. tularensis or Y. pestis in the BALB/c mouse. This is to determine how effective finafloxacin may be if used in infected but asymptomatic people compared to patients displaying symptoms, and provides proof-of concept efficacy data for these biothreat pathogens. In these studies, prophylaxis was initiated at 24 h postchallenge, with treatment administered at 72 h postchallenge for F. tularensis or at 38 h postchallenge for Y. pestis. The differing schedules were due to differences in the progression of disease in the BALB/c mouse model. In addition, a short course of therapy (3 days) was compared to a longer course (7 days).

## RESULTS

### Efficacy against an infection with F. tularensis.

The efficacy of finafloxacin was compared to ciprofloxacin and controls treated with the vehicle (Tris buffer or phosphate-buffered saline [PBS]) following an inhalational infection with F. tularensis. Mice were challenged with a mean retained dose of 272 CFU (range, 245 to 308 CFU) equating to approximately a 50% lethal dose (LD_50_) of 54 (LD_50_ is <5 CFU). At 24 h postchallenge, mice were not displaying clinical signs of disease; by 72 h postchallenge, they were showing mild to moderate signs of disease. All control animals (untreated or treated with the vehicle or PBS) succumbed to infection by day 4 postchallenge. Ten animals per treatment group were observed for survival until the experiment was terminated on days 34 and 35 postchallenge (due to the high numbers of survivors).

**FIG 1:**
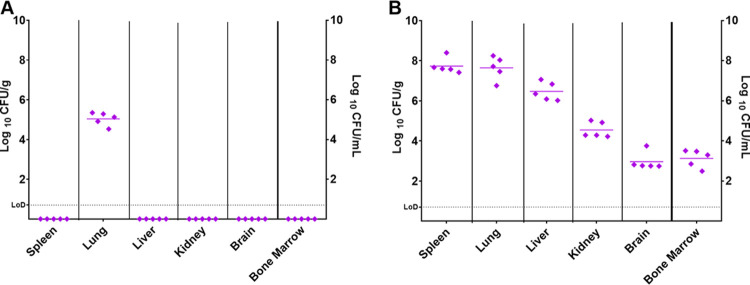
Concentration of F. tularensis in organs before treatment was initiated. Bacterial counts (CFU/g of tissue or CFU/ml bone marrow) were determined in panels of organs at 24 or 72 h postchallenge. (A) Five mice were challenged and euthanized at 24 h postchallenge. (B) Five mice were challenged and euthanized at 72 h postchallenge. LoD, limit of detection.

Before treatment was initiated, at either 24 or 72 h postchallenge, groups of 5 animals were euthanized to investigate the progression of disease. The spleen, liver, lungs, brain, kidneys, and bone marrow were harvested and processed for bacterial burden. Sections of the liver, lungs, and spleen were also collected for histopathological analysis to identify structural changes in the tissues or evidence of residing bacteria. At 24 h postchallenge, F. tularensis was detected in all of the lung samples (ranging from 3.4 × 10^4^ to 2.2 × 10^5^ CFU/g). All other organs were clear from bacteria, suggesting that early in the infection, the bacteria remain within the primary site (lung) (Fig. 1A). Histopathological changes were also restricted to the lung, where minimal areas of acute neutrophilic infiltration were seen in the alveoli of 2 mice.

At 72 h postchallenge, F. tularensis was detected in all of the organs harvested, with the highest concentrations observed in the spleen (2.6 × 10^7^ to 2.5 × 10^8^ CFU/g) and lung (5.7 × 10^6^ to 1.8 × 10^8^ CFU/g) (Fig. 1B). Histopathological changes of acute multifocal neutrophilic and necrotizing inflammation consistent with F. tularensis infection were observed in the lungs, spleen, and liver of the infected mice at 72 h. There were no differences in the weights of the organs from mice that were euthanized at 24 or 72 h postchallenge (data not shown).

**FIG 2:**
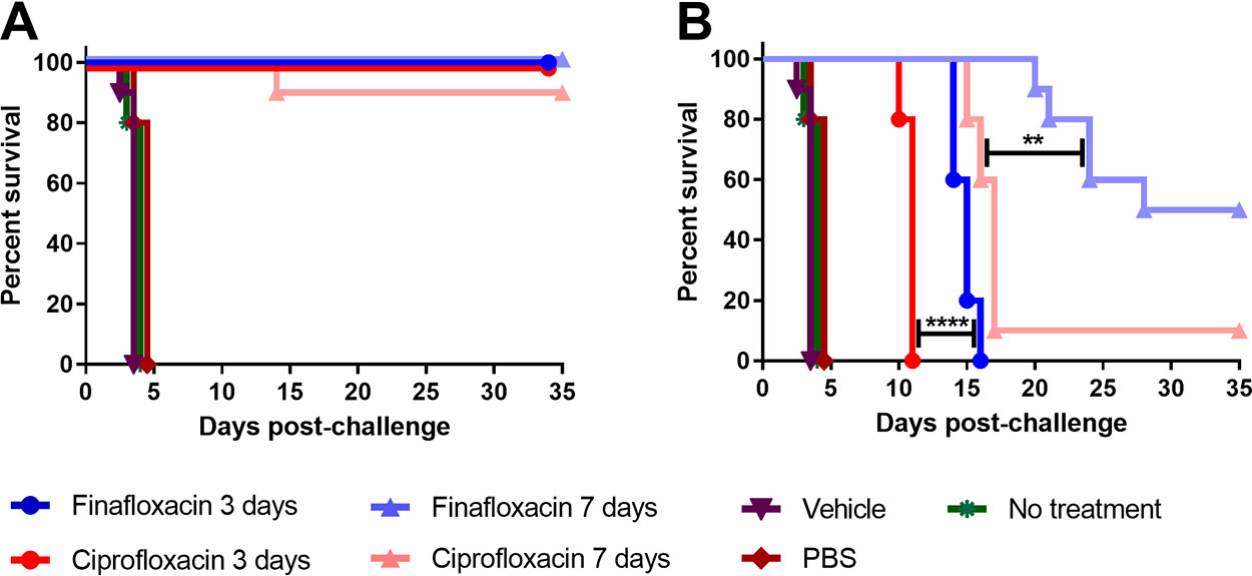
Percentage survival of mice in each treatment group following challenge with aerosolized F. tularensis. Mice were challenged with a mean retained dose of 272 CFU of F. tularensis by the inhalational route and treated with finafloxacin (23.1 mg/kg) every 8 h by the oral route or ciprofloxacin (30 mg/kg) every 12 h by the i.p. route. Control animals received substances by the oral (vehicle) or i.p. (PBS) route. Regimens were initiated at 24 (A) or 72 (B) hours postchallenge for 3 or 7 days. **, *P* < 0.01; ****, *P* < 0.0001.

When therapy was initiated at 24 h postchallenge, both finafloxacin and ciprofloxacin offered significant protection in comparison to the untreated or PBS- or vehicle-treated controls (*P* < 0.0001), irrespective of the length of the dosing regimen (Fig. 2). There was no significant difference between the antibiotic-treated groups (*P* > 0.05) (Fig. 2A). When therapy was initiated at 72 h postchallenge and continued for 3 days, all of the mice succumbed to disease; however, there was a significant increase in time to death for the mice treated with finafloxacin (*P* < 0.0001) in comparison to the mice treated with ciprofloxacin (Fig. 2B). Both antibiotics offered significant protection in comparison to the controls (*P* < 0.0001). When therapy was initiated at 72 h postchallenge and continued for 7 days, finafloxacin provided a higher level of protection (50%) than mice treated with ciprofloxacin (10%) (*P* < 0.01). Organs were harvested from all surviving animals and processed (as described above). All surviving animals were clear from colonizing bacteria, and no histopathological changes typical of an infection with F. tularensis were observed in tissues.

As body weight data are difficult to analyze once animals start to succumb to disease, the following inclusion criteria have been used for analysis: where a comparator group has lost greater than 10% of its participants, no further comparisons will be made of that group. There was evidence of a treatment effect on weight change for all regimens over the course of the study when finafloxacin was compared to ciprofloxacin (Fig. S1 in the supplemental material; *P* < 0.001 in all cases). However, the effect was minimal and was not easy to determine at individual time points, apart from those receiving treatment for 3 days initiated at 72 h postchallenge when additional statistical tests were utilized (Fig. S1C). Mice treated with finafloxacin lost less weight than those treated with ciprofloxacin, which was identifiable at days 9 to 10 postchallenge. Comparisons from 10 days postchallenge onward in these mice were not made, as too many mice had succumbed to infection.

**FIG 3:**
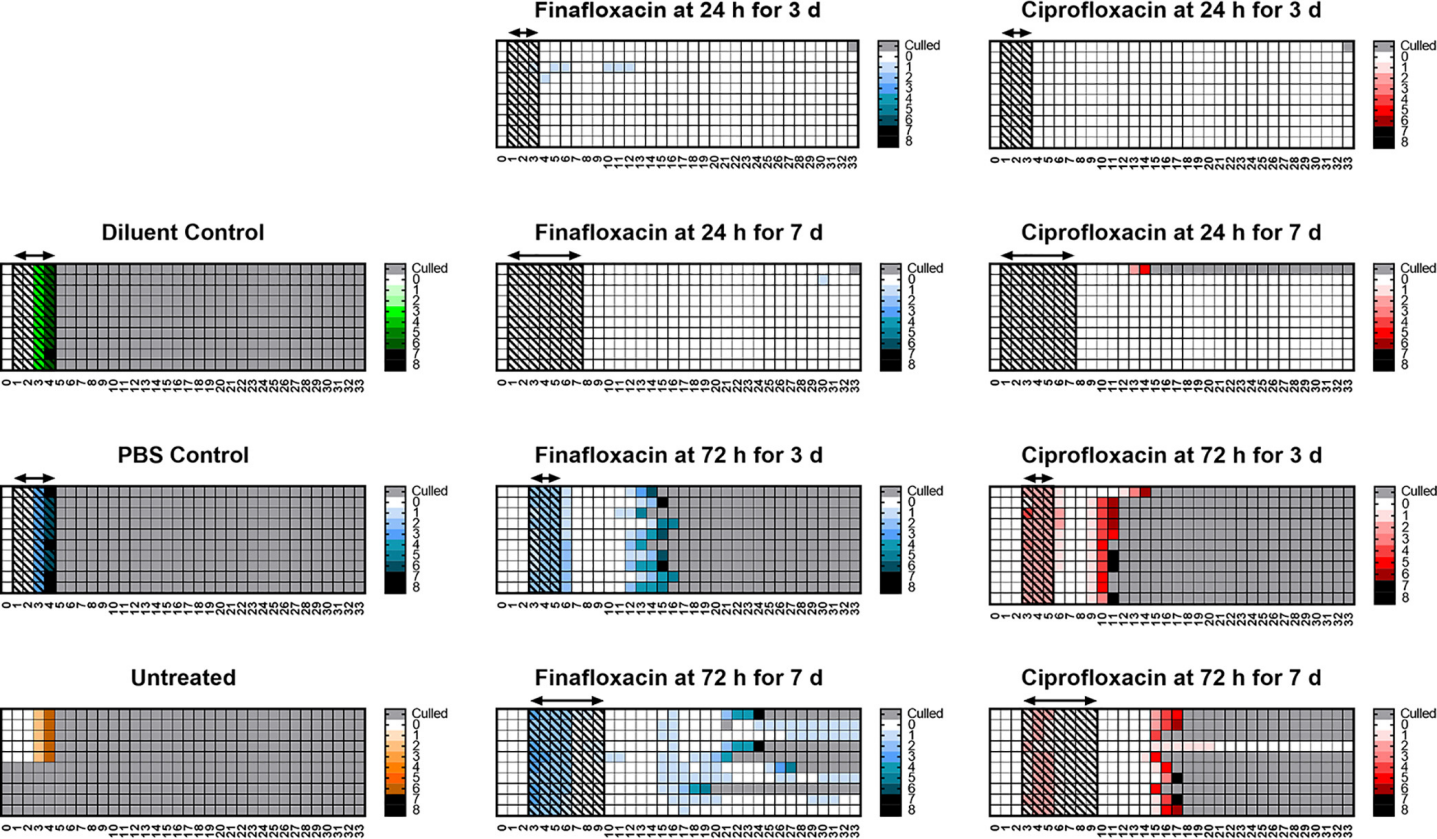
Clinical scores recorded throughout the study. Mice were challenged with a mean retained dose of 272 CFU of F. tularensis by the inhalational route and treated with finafloxacin (23.1 mg/kg) every 8 h by the oral route or ciprofloxacin (30 mg/kg) every 12 h by the i.p. route. Control animals received substances by the oral (vehicle) or i.p. (PBS) route. Regimens were initiated at 24 or 72 h postchallenge and continued for 3 or 7 days. These are heat maps for each mouse in the study, determined using the clinical scores recorded for each individual mouse, a minimum of twice daily. The lighter the color, the less severe the disease. Gray boxes represent animals that succumbed to infection. The arrows and shaded boxes represent the treatment period.

The majority (90%) of the mice treated at 24 h postchallenge did not develop signs of disease, compared to those that were treated later in the infection; all surviving mice had no clinical signs of disease at the end of the study. All mice treated at 72 h postchallenge developed signs of disease. Those treated for 3 days at 72 h postchallenge recovered following the initiation of treatment and appeared to resolve the disease; however, following cessation of treatment, they relapsed with infection. The mice receiving finafloxacin had resolved all clinical scores by day 7 and started to develop clinical signs by day 11, and by day 16, all had succumbed to disease. However, 20% of the mice that received ciprofloxacin did not resolve their clinical signs, 80% showed no signs for 2 days at day 9, and all had succumbed to disease by day 11. Thirty-three percent of the surviving mice were not displaying signs of disease at the end of the study. Mice receiving ciprofloxacin for 3 days initiated at 72 h postchallenge had higher scores than mice receiving finafloxacin for the same regimen. The actual scores recorded for individual mice are shown in the heat maps in Fig. 3.

### Efficacy against an infection with Y. pestis.

The efficacy of finafloxacin was compared to ciprofloxacin and controls treated with the vehicle (Tris buffer or PBS) following an inhalational infection with Y. pestis. Mice were challenged with a mean retained dose of 8.9 × 10^3^ CFU (range, 5 × 10^3^ to 1.2 × 10^4^) equating to an LD_50_ of approximately 14 (LD_50_, approximately 600 CFU). At 24 h postchallenge, mice were not displaying clinical signs of disease; by 38 h postchallenge, 35% of the mice in the treatment groups were showing signs, which ranged from mild to severe. All control animals (untreated or treated with the vehicle or PBS) succumbed to infection by day 4 postchallenge. Ten animals per treatment group were observed for survival until the experiment was terminated on days 35, 36, and 37 postchallenge (due to the high number of survivors).

**FIG 4:**
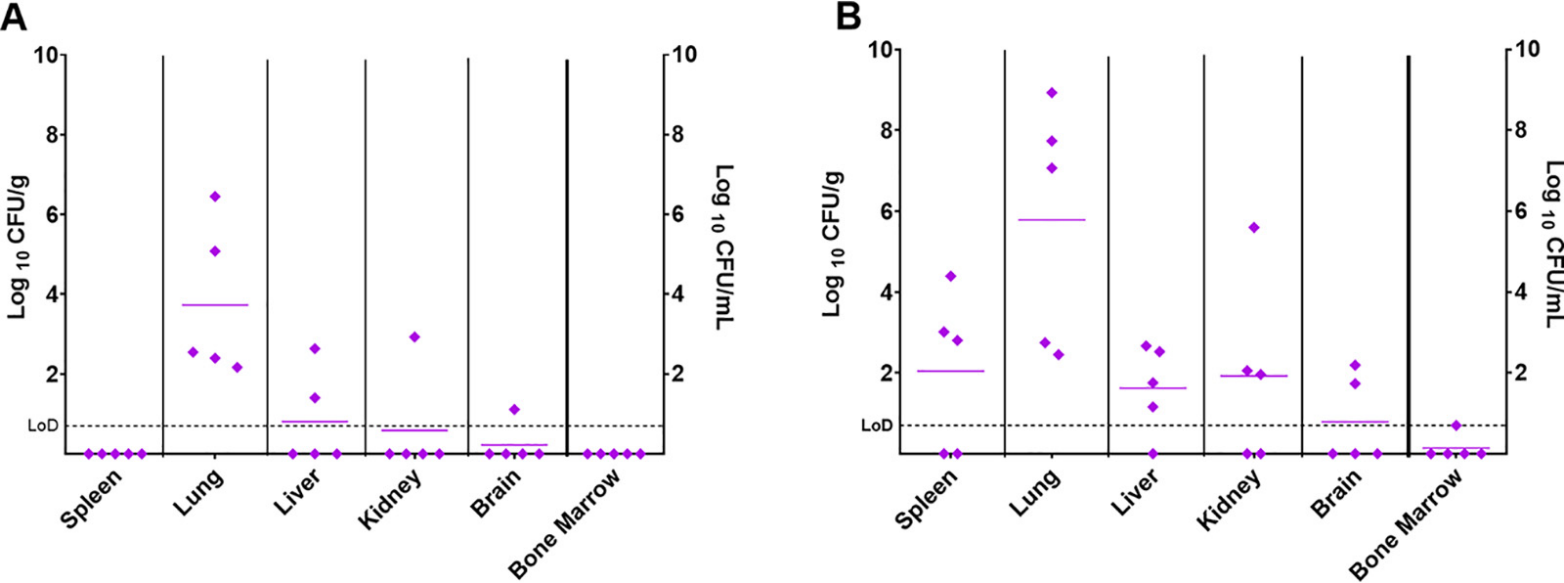
Concentration of Y. pestis in organs before treatment was initiated. Bacterial counts (CFU/g of tissue or CFU/ml) were determined in panels of organs at 24 or 38 h postchallenge. (A) Five mice were challenged and euthanized at 24 h postchallenge. (B) Five mice were challenged and euthanized at 38 h postchallenge. LoD, limit of detection.

Before treatment was initiated at either 24 or 38 h postchallenge, groups of 5 animals were euthanized to investigate the progression of disease. The spleen, liver, lungs, brain, kidneys, and bone marrow were harvested and processed for bacterial burden. Sections of the liver, lungs, and spleen were also collected for histopathological analysis. At 24 h postchallenge, Y. pestis was detected in all of the lung samples (levels ranging from 1.5 × 10^2^ to 2.8 × 10^6^ CFU/g). One mouse had low levels in the liver, kidney, and brain, with an additional mouse colonized in the liver. These two mice had the highest bacterial load in the lung. All other organs were clear from bacteria, suggesting that early in the infection, the bacteria generally remain within the lung (Fig. 4A). No significant histopathological changes were seen at this time point. At 38 h postchallenge, the distribution of detectable Y. pestis was very variable. All mice were colonized in the lung (ranging from 2.8 × 10^2^ to 8.5 × 10^8^ CFU/g); again, the mice heavily colonized in the lungs were also colonized in the other organs (Fig. 4B). One mouse was only colonized in the lung, with all other organs clear from detectable bacteria. Histopathological changes were only observed in two of the mice and limited to the lung as an acute neutrophilic bronchopneumonia with occasional areas of necrosis. There were no differences in the weights of the organs from mice that were euthanized at 24 or 38 h postchallenge (data not shown).

**FIG 5:**
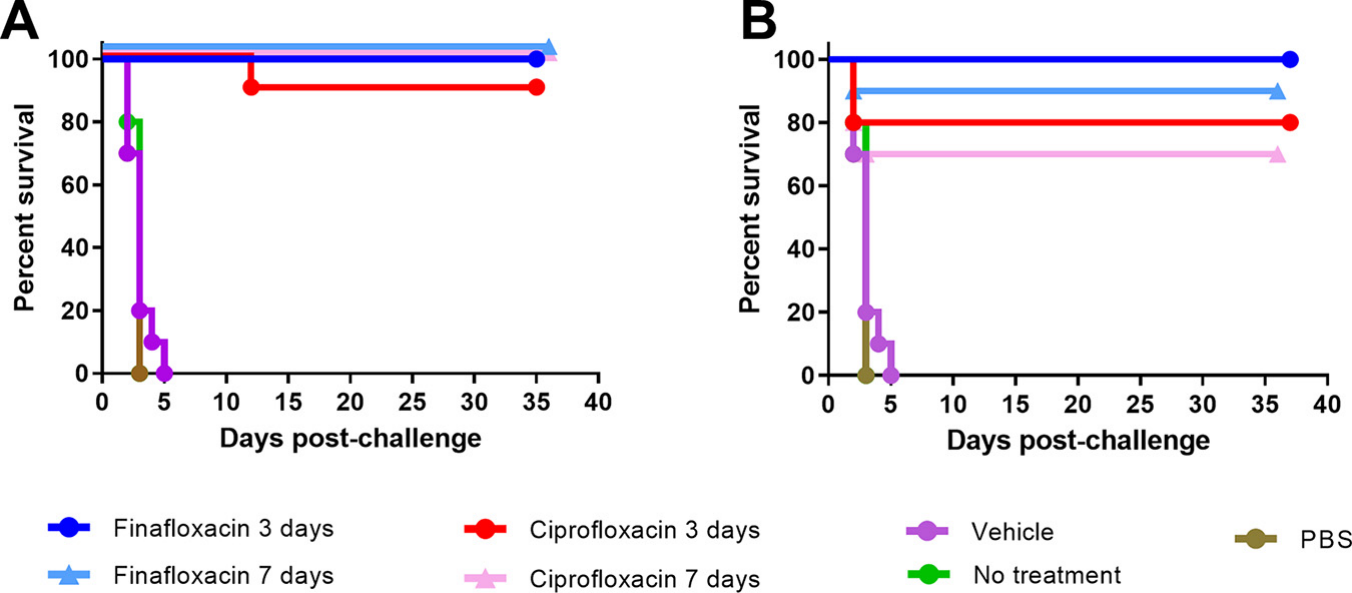
Percentage survival of mice in each treatment group following challenge with aerosolized Y. pestis. Mice were challenged with a mean retained dose of 8.9 × 10^3^ CFU of Y. pestis by the inhalational route and treated with finafloxacin (23.1 mg/kg) every 8 h by the oral route or ciprofloxacin (30 mg/kg) every 12 h by the i.p. route. Control animals received substances by the oral (vehicle) or i.p. (PBS) route. Regimens were initiated at 24 h postchallenge for 3 or 7 days (A) or at 38 h postchallenge for 3 or 7 days (B).

When therapy was initiated at 24 or 38 h postchallenge, both finafloxacin and ciprofloxacin offered significant protection in comparison to the untreated or PBS- or vehicle-treated controls (*P* < 0.0001), irrespective of length of dosing regimen (Fig. 5A and B). When individually analyzed across each time of initiation and duration of therapy, there were no significant differences between the antibiotic-treated groups (*P* > 0.05). All surviving animals were clear from colonizing bacteria with no significant pathological changes typical of an infection with Y. pestis observed in tissues, with the exception of one mouse (treated at 38 h postchallenge with 3 days of finafloxacin) (data not shown).

Changes in mouse body weight were recorded and analyzed as a measure of health (Fig. S2). There was evidence of a treatment effect on weight change for all treatment groups (*P* < 0.001 in all cases) over the course of the study, except the group receiving treatment at 38 h postchallenge for 3 days (*P* = 0.9534). Again, the effect was minimal, and the exact nature of the differences was not easy to determine, apart from those receiving treatment for 7 days initiated at 38 h postchallenge, when additional statistical tests were utilized (Fig. S2B). Mice treated with ciprofloxacin lost less weight than those treated with finafloxacin, identifiable at days 2, 5, 6, and 7 postchallenge.

**FIG 6:**
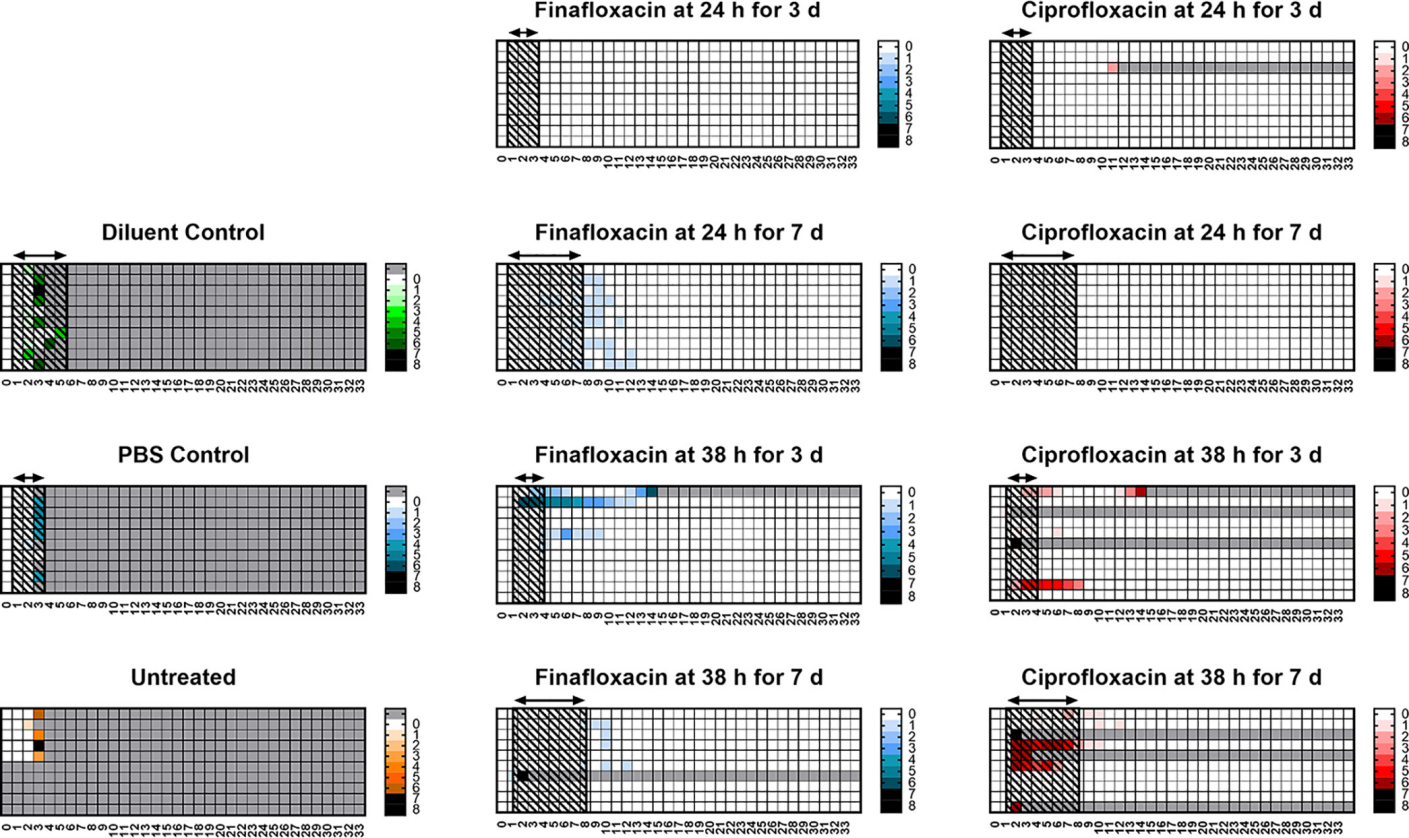
Clinical scores recorded throughout the study. Mice were challenged with a mean retained dose of 8.9 × 10^3^ CFU of Y. pestis by the inhalational route and treated with finafloxacin (23.1 mg/kg) every 8 h by the oral route or ciprofloxacin (30 mg/kg) every 12 h by the i.p. route. Control animals received substances by the oral (vehicle) or i.p. (PBS) route. Regimens were initiated at 24 or 38 h postchallenge and continued for 3 or 7 days. These are heat maps for each mouse in the study, determined using the clinical scores recorded for each individual mouse, a minimum of twice daily. The lighter the color, the less severe the disease. Gray boxes represent animals that succumbed to infection. The arrows and shaded boxes represent the treatment period.

The clinical scores data showed that mice in the control groups had short periods of declining health until they succumbed to infection; however, only 48% of the antibiotic-treated mice showed any clinical signs of infection. Mice receiving finafloxacin for 7 days, initiated at 24 h postchallenge, had higher scores than the comparator group who received ciprofloxacin. Eighty percent of the mice receiving finafloxacin scored mild signs of disease (scores of 1) during the treatment phase until days 12 to 13 postchallenge, which is likely to be due to the effects of the antibiotic. Similarly, 20% of the ciprofloxacin-treated mice were reported to have low scores, again resolving by day 12 postchallenge. The actual scores recorded for this regimen are shown in the heat maps in Fig. 6.

## DISCUSSION

Alternative therapies for the treatment of tularemia and plague are warranted, and antibiotics that can be delivered orally would have utility in reducing the burden associated with delivering antibiotics intravenously within a hospital setting. The fourth-generation fluoroquinolone finafloxacin offers advantages to traditional antibiotics. Finafloxacin (as other fluoroquinolones) is available in multiple formulations, which would allow for self-administration or for a patient to be treated initially with an i.v. formulation in hospital followed by oral step-down treatment if required. This approach has been shown to be successful in clinical trials treating patients with complicated UTIs (31). These human trials also demonstrated that a shorter course of finafloxacin treatment resulted in a better outcome than a longer course of ciprofloxacin. The use of shorter courses of antibiotics offers advantages, including increasing the likelihood of antibiotic regimens being completed and a reduction in the development of potential side effects and antimicrobial resistance.

The proof-of-concept studies detailed in the manuscript demonstrate that, irrespective of the bacterial agent, treating early in an infection results in a better outcome. All dosing regimens offered a high level of protection when administered at 24 h postchallenge to mice infected with F. tularensis. This aligns with published data that reported complete protection when ciprofloxacin or finafloxacin were delivered for 7 days, initiated at 24 h postchallenge to mice intranasally infected with F. tularensis (35, 36). Although the route of infection was different, the LD_50_ of strain SCHU S4 by both routes is low (<10 CFU) (37, 38). In this F. tularensis study, no differences were observed between the therapies, suggesting that when the infection remains within the lung, it is easier to treat successfully. When the therapies were delivered later in the infection (at 72 h postchallenge, when the mice were showing clinical signs of disease and bacteria had disseminated throughout the animal), then differences between the treatments were observed. Finafloxacin provided a protective benefit compared with ciprofloxacin when delivered for 3 days (resulting in an increase in time to death of 5 days) or 7 days (an improved level of protection). This was also demonstrated in the intranasal model where finafloxacin and ciprofloxacin offered 96% and 60% protection, respectively, when delivered for 7 days and initiated at 72 h postchallenge (35).

The increase in time to death observed when finafloxacin is delivered for 3 days initiated at 72 h postchallenge demonstrates a significant advantage to ciprofloxacin, one of the currently recommended treatments for tularemia (16). The animals treated with ciprofloxacin were completely protected until 4 to 5 days following cessation of the antibiotic; they then succumbed to disease, compared to those treated with finafloxacin that started to succumb to infection at day 14 postchallenge. This delay in time to death was also associated with lower clinical signs and less body weight loss than groups receiving ciprofloxacin. Treating at 72 h postchallenge for longer (7 days) demonstrated further utility for finafloxacin as an alternative treatment for tularemia. Again, those mice treated with finafloxacin were completely protected for longer (until day 21 postchallenge) than those treated with ciprofloxacin (until day 16 postchallenge). Half of the mice treated with finafloxacin survived until the end of the study compared to 10% of those treated with ciprofloxacin. The organs from survivors were clear of colonizing bacteria, and there was no evidence of pathological changes in the tissues.

Relapse of infection with F. tularensis that is associated with a delay in treatment initiation is well documented in mouse models and has been reduced with longer dosing regimens (36). Treating the mice for longer may have better controlled the infection and resulted in an improvement in survival offered in this study. The BALB/c mouse is also very sensitive to infection with F. tularensis. There is a balance required in demonstrating high levels of protection in animal models and using dosing regimens utilized to treat human infections. Alternatively, demonstrating a protective benefit with fewer doses of the antibiotic would also be advantageous. This encouraging data also align with data generated in an intranasal infection of F. tularensis. Again, finafloxacin demonstrated the ability to protect mice from infection when delayed for up to 96 h postchallenge, with no animals succumbing to infection following cessation of the treatment (35).

For Y. pestis, all dosing regimens offered a high level of protection when administered at 24 h postchallenge, with no differences observed between the therapies and no colonizing bacteria detected in the organs of surviving animals. This aligns with previously published data with the comparator fluoroquinolone, ciprofloxacin, that reported 100% protection following 3 and 5 days of therapy, respectively, initiated at 24 h postchallenge (39, 40). Interestingly, although there was no evidence of residual bacteria or pathological changes associated with Y. pestis infection in survivors in this study, those animals treated with finafloxacin had average clinical scores that were higher than the ciprofloxacin comparator (when treated at 24 h postchallenge for 7 days), although the scores were very low. We hypothesize that this is a transient effect of the antibiotic, apparent when administered for a longer period of time. By day 12 to 13 postchallenge, no further scores from this group were observed.

At 38 h postchallenge, some mice were showing clinical signs of disease (ranging from mild to severe). Again, all treatment regimens offered high levels of protection, and when the data were combined, finafloxacin offered a significant benefit to ciprofloxacin. In addition, the mice that received finafloxacin for 7 days lost more weight than those treated with ciprofloxacin; however, 3 days of finafloxacin delivered at 38 h postchallenge was able to clear the active infection (although not the chronic lesions in one animal), resulting in this animal recovering from disease. This aligns well with similar observations from the F. tularensis study. It is worth stating, however, that there was a lot of variability in the bacterial load between individual animals observed before the treatment was initiated. It is unclear why this variation was observed; however, it did not translate into differences in survival. This may be due to how the infection is established in the mouse model and how the bacteria is able to disseminate in individual animals. It has been very well documented that unlike F. tularensis (which is more of an intracellular organism), Y. pestis is able to disseminate from an intracellular niche (when it first gains entry to host cells) to an extracellular niche with antiphagocytic resistance (when it infects the lymph node) (41, 42). It is possible that the environment created by the immune response fighting the infection may have resulted in an environment of lower pH that would be advantageous to finafloxacin. Finafloxacin is likely to have activity in both arenas, whereas ciprofloxacin is likely to be less effective in an intracellular, more acidic environment (28, 33). This may account for some of the subtle differences between the efficacy of finafloxacin in the two models and may explain why 50% of the animals treated with 7 days of finafloxacin “recovered” from infection with F. tularensis when initiated late (at 72 h postchallenge).

Again, the high levels of protection observed in this study following antibiotic treatment (70% in all cases) are similar to published data generated in previous studies. Delivering ciprofloxacin at 42 h postchallenge for 5 days resulted in 50% protection (40). Initiating antibiotic treatment later on in the disease (42 to 48 h postchallenge) does result in a reduction in the level of protection; therefore, better discrimination between the therapies may be observed in the future if the treatment window was further extended and if there is more severe disease (43).

The data generated in these studies provide further evidence of how finafloxacin could be used as an alternative to ciprofloxacin as a prophylaxis or to treat infections with F. tularensis or Y. pestis. The availability of finafloxacin in multiple formulations (as the other fluoroquinolones, including ciprofloxacin) would allow this antibiotic to be administered in hospitals to very ill patients or be self-administered by the patient, reducing the cost of expensive hospital stays. This would be very important if either of these bacterial agents were deliberately released. As finafloxacin has also demonstrated the ability to protect mice against B. pseudomallei and has *in vitro* activity against larger panels of the biothreat agents, it should be considered an alternative broad-spectrum antibiotic for the treatment of all infections of this nature (33, 34).

## MATERIALS AND METHODS

### Bacteria.

All work with F. tularensis and Y. pestis was carried out within fully contained class III microbiological safety cabinets within a biosafety level 3 (BSL3) laboratory in facilities that are registered and inspected by the UK Health and Safety Executive, as required under the Control of Substances Hazardous to Health (COSHH) regulations, and all associated guidance, for BSL3 pathogens. These pathogens were handled in accordance with safety documentation that was reviewed by the Biological Safety Risk Committee and the Biological Safety Officer and authorized by management.

F. tularensis strain SCHU S4 was streaked onto blood cysteine glucose agar (BCGA) plates and incubated at 37°C overnight. The following day, a loopful of bacteria was inoculated into 5 ml phosphate-buffered saline (PBS) and adjusted to an optical density at 590 nm (OD_590_) of 0.2. One milliliter of this was used to inoculate 100 ml modified cysteine partial hydrolysate (MCPH) broth supplemented with cysteine (100 μg/ml) and glucose (4%, wt/vol) and incubated with shaking at 37°C for 48 h. The OD_590_ of the culture was adjusted to 0.1, which equates to approximately 1 × 10^8^ CFU/ml. A dilution of 1 in 75 was performed, and 15-ml aliquots were used in the challenge nebulizer.

Y. pestis strain CO92 was grown for 48 h at 28°C on blood agar base (BAB) plates supplemented with 0.02% hemin. The following day, a loopful of bacteria was harvested into 5 ml PBS and adjusted to an OD_590_ of 0.5. A 1-ml aliquot was inoculated into 50 ml BAB broth and incubated for 40 h at 28°C, with shaking at 180 rpm. The culture was diluted 1 in 5, and 15-ml aliquots were used in the challenge nebulizer.

### Mice.

Animal studies were carried out in accordance with the UK Animals (Scientific Procedures) Act 1986, a UK Home Office project license and an Animal Care and Use Review Office (ACURO) appendix. Female BALB/c mice (Charles River Laboratories, UK) aged 8 to 10 weeks were randomized into cages of 5 and housed within a class III half-suit rigid isolator in an Advisory Committee on Dangerous Pathogens (ACDP) containment level 3 laboratory. Mice had free access to water and rodent diet (Harlan Teklad, UK) and underwent a period of acclimatization for at least 5 days before any procedures were performed.

### Antibiotics.

Finafloxacin in salt form was supplied by MerLion Pharmaceuticals Ltd. A 15-mg/ml solution of finafloxacin was prepared by adding 2.1 ml of 0.01 M Tris buffer to 44 mg of finafloxacin powder (containing 37.5 mg of active ingredient). We added 200 μl of 1 M sodium hydroxide to dissolve the antibiotic, followed by 200 μl of 0.01 M hydrochloric acid. Ciprofloxacin (Ciproxin solution for infusion; Bayer, UK) was used at a stock concentration of 2 mg/ml.

### Efficacy studies.

In the first study, animals were challenged by the aerosol route with F. tularensis. Mice were restrained within a nose-only exposure tube and placed within an exposure chamber. Bacteria were aerosolized using a Collison three-jet nebulizer within a contained Henderson apparatus controlled by the AeroMP (aerosol management platform) (Biaera Technologies LLC). Fifteen milliliters of bacteria were placed into the nebulizer, and the mice were exposed for 10 min to a dynamic aerosol conditioned by the AeroMP. The aerosol stream was maintained at 50 to 55% relative humidity and 22°C. The concentration of F. tularensis in the aerosol was determined by recovering samples from the exposure chamber using an all-glass impinger (AGI30) operating at 12 liters/min, containing 10 ml of sterile PBS. Impinger samples were plated out onto BCGA, and the retained dose of bacteria that mice received in each run was calculated by applying the Guyton formula (44). It was assumed that each mouse retained 40% of the organisms that were inhaled (45).

In a separate study, animals were challenged by the aerosol route with Y. pestis. For this study, the aerosol stream was maintained at 40% relative humidity, and the impinger samples were plated out onto BAB plates. All other conditions were the same as previously described.

Treatment was initiated at 24 or 72 h (F. tularensis) or 24 or 38 h (±2 h) (Y. pestis) postchallenge and continued for 3 or 7 days. Groups of 10 mice were administered finafloxacin (23.1 mg/kg) or the vehicle control (Tris-buffered saline) in a 31-μl oral dose via pipette every 8 h. Ciprofloxacin (30 mg/kg) or PBS was administered every 12 h by the intraperitoneal (i.p.) route. These regimens were determined following pharmacokinetic studies and aimed to deliver a human-equivalent dose (Table S1 in the supplemental material). In each study, one group of animals was infected and untreated. Mice were weighed daily and observed a minimum of twice daily for clinical signs of disease (e.g., piloerection, hunching, respiratory problems, eye problems, mobility problems) until the experiment was terminated.

At the initiation of therapy, 5 mice that were infected but left untreated were euthanized. Postmortems were performed on all mice and organs harvested and weighed and tissue samples collected. Sections of the spleen, liver, and lung were fixed in 10% neutral buffered formalin (NBF) and submitted to the Animal and Plant Health Agency (APHA) for histopathological examination. Samples were blocked into histology cassettes and processed to wax blocks. Four-micrometer-thick sections were cut using a rotary microtome and stained with hematoxylin and eosin (H&E) for analysis.

The remaining tissues were homogenized in 1 ml of PBS, and 100-μl aliquots were plated onto BCGA plus LCAT (lincomycin, colistin sulfate, amphotericin B, and trimethoprim) (F. tularensis) or BAB (Y. pestis) agar plates in duplicate. The plates were incubated for 3 days at 37°C (F. tularensis) or 2 days at 28°C (Y. pestis) and enumerated to determine the bacterial load in the organs. The remaining homogenate was placed into liquid media and incubated for 5 days. For organs that were clear by agar plating, a 10-μl aliquot of the relevant incubated homogenate was streaked onto agar plates and incubated for a further 3 days to identify any further F. tularensis or Y. pestis colonies. At the end of these studies, all surviving mice were culled and the organs harvested and processed as described above.

### Statistical analysis.

Graphs were prepared using PRISM v6.0, 7.0, or 8.0 (GraphPad). Survival data were compared using log rank tests using SPSS v21.0 (IBM) and, on one occasion, by Cox regression using R. Where stipulated, some of the log rank tests were stratified by factors such as treatment time.

Changes in body weight were analyzed using the software GraphPad Prism v8.0. Weight data were transformed to a percentage compared to baseline. As mice succumbed to infection, these data became censored. Mice that succumbed close to the onset of treatment were not considered. At time points where less than 90% of the original cohort were available, analysis ceased. This arbitrary threshold was used, as the most severely ill mice will succumb to infection, thus reducing the difference between groups and removing the likelihood of false positives.

Weight change profiles were compared using linear models, where the mouse was a repeated measurement and time was included as a categorical factor. Given that the data set started from a position of no difference (until therapy was initiated), the interaction component of the model is used as a measure of difference. The assumptions of the test were assessed using residual plots and QQ plots. The test requirements for homoscedasticity and Gaussian distribution were reasonable assumptions with a small number of negative outlier values. Sidak’s multiple comparisons were used to locate when mice receiving treatment were likely to have different weights related to starting weight. Due to the large numbers of time points, these posttests had little statistical power.
