# Supplementary material for: Finafloxacin Is an Effective Treatment for Inhalational Tularemia and Plague in Mouse Models of Infection
Source: Antimicrob Agents Chemother. 2021 May 18;65(6):e02294-20. doi: 10.1128/AAC.02294-20 (PMC8315961; doi:10.1128/AAC.02294-20)
Supplement: Supplementary file 1 [file aac.02294-20-s0001.pdf]

Table S1. The non-compartmental PK parameters determined for ciprofloxacin and finafloxacin in the plasma of Balb/C mice.

Antibiotic	Species	Total administered Dose	Tissue	Cmax (µg/ml)	AUC (µg·h/ml)	T½ (h)	CL (l/h/kg)
**Ciprofloxacin**	Mouse (Balb/c)	30 mg/kg (i.p.) (1)	Plasma	16.9	11.6	1.1	2.59
	Human	500 mg (p.o.) (2)	Serum	2.7 ± 0.8	10.7 ± 2.6	5.7 ± 1.2	≈0.51*
**Finafloxacin**	Mouse (Balb/c)	SIMULATED† 23.1 mg/kg (p.o.) (3)	Plasma	4.2	24.0	1.2	2.9
	Human	800 mg (p.o.) Day 7 data (4)	Plasma	8.95 ± 3.11	26.1 ± 8.6	14.0 ± 5.5	0.39 ± 0.13‡

*Data normalised to a nominal bodyweight of 70 kg.

The ciprofloxacin data was previously published in (1).

†The pharmacokinetic parameters were predicted by simulation using data from a higher dose of 37.5 mg/kg (p.o.) (3).

‡ Data normalised to the mean bodyweight reported in (4).

The mean ciprofloxacin and finafloxacin concentration-time profile was generated from the plasma concentrations and PK the analysis of these profiles was completed using Phoenix WinNonlin v 8.0 (Certara Inc) to calculate parameters including the maximum drug concentration (C_{max}), the area under the curve, (AUC), clearance (CL), and the terminal half-life (T½)

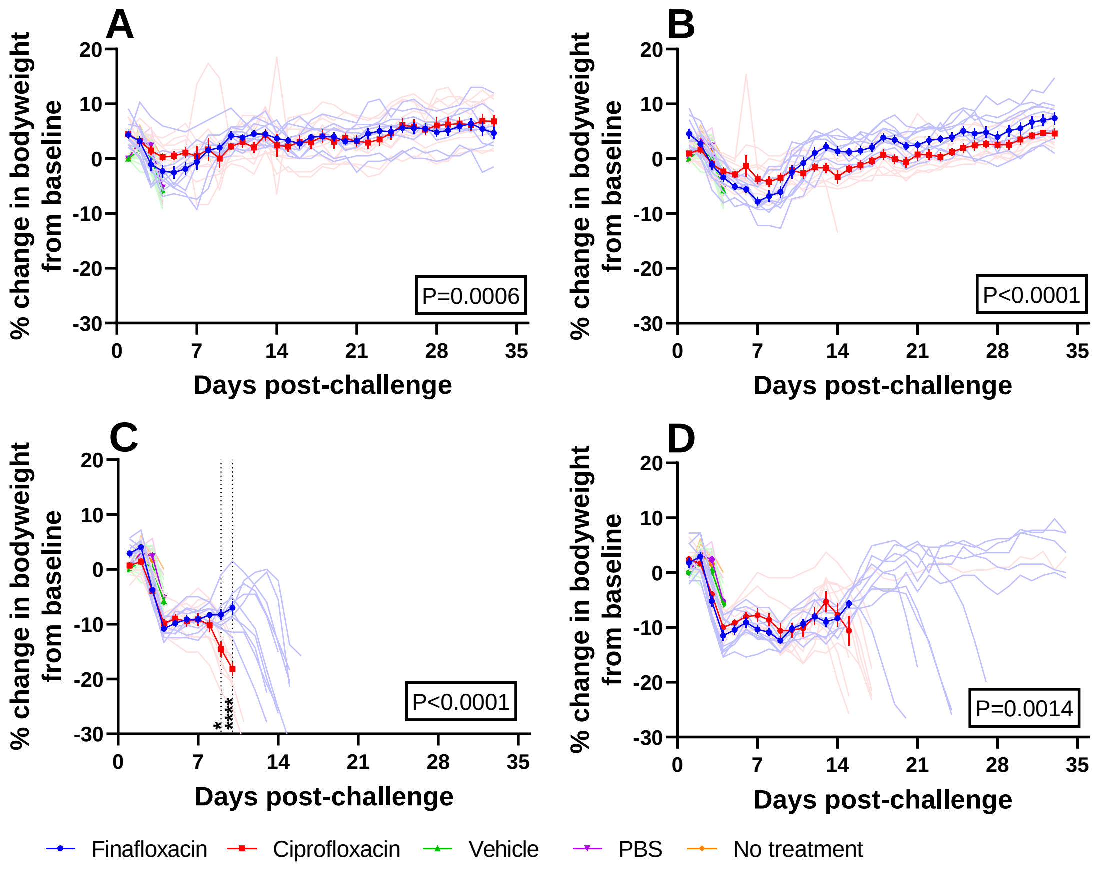

Figure S1. The bodyweight profile recorded throughout the study. Mice were challenged with a mean retained dose of 272 CFU of *F. tularensis* by the inhalational route and treated with finafloxacin (23.1 mg/kg) every 8 hours by the oral route or ciprofloxacin (30 mg/kg) every 12 hours by the IP route. Control animals received substances by the oral (vehicle) or IP (PBS) route. Regimens were initiated at 24 hours post-challenge and continued for 3 (A) or 7 (B) days or initiated at 72 hours post-challenge and continued for 3 (C) or 7 (D) days. The faint lines are the weights of individual animals recorded throughout the study and the thick lines are the means of the groups ± the standard error of the mean (SEM). The data was analysed by the linear model and the p values shown are the differences between the treatments over time. In addition, Bonferroni's multiple comparisons were used to compare groups at specific time points. * p<0.05 **** p<0.0001.

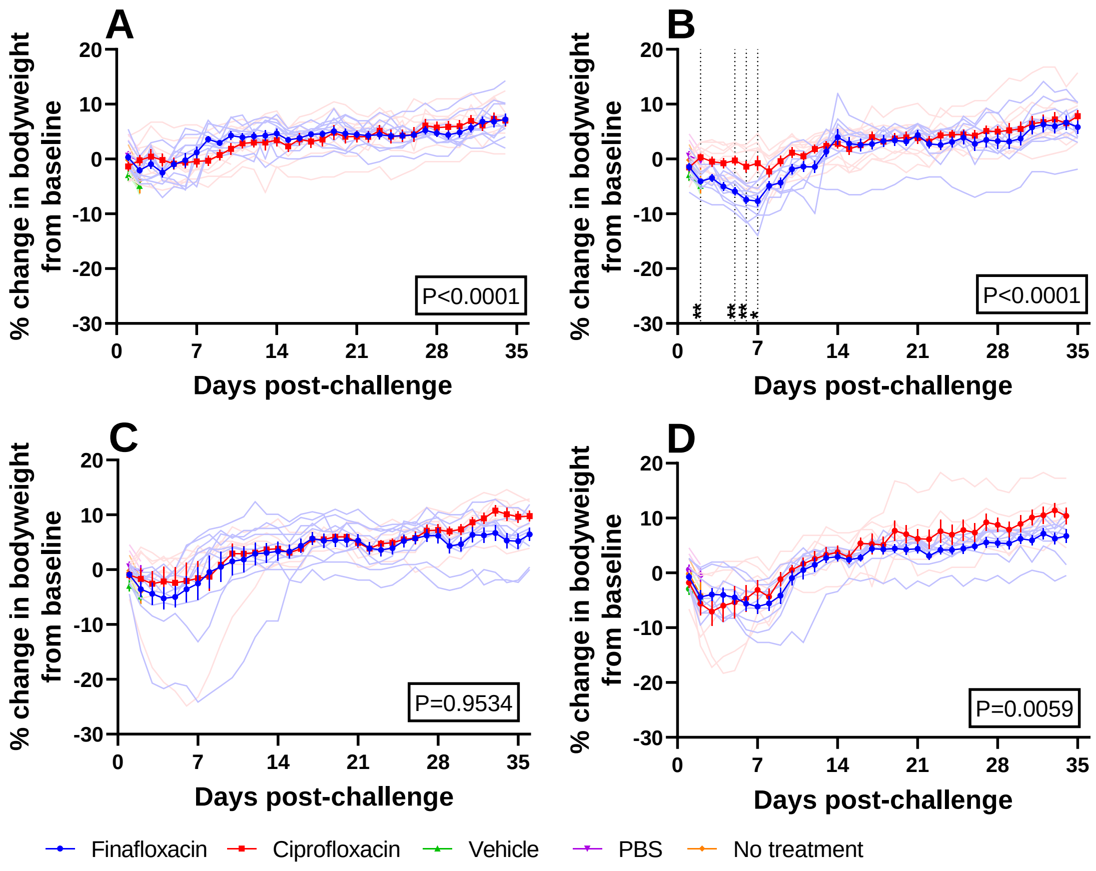

Figure S2. The weight loss profile of mice treated at 24 or 38 hours post-challenge with *Y. pestis*. Mice were challenged with a mean retained dose of 8.9 x 10^3 CFU of *Y. pestis* by the inhalational route and treated with finafloxacin (23.1 mg/kg) every 8 hours by the oral route or ciprofloxacin (30 mg/kg) every 12 hours by the IP route. Control animals received substances by the oral (vehicle) or IP (PBS) route. Regimens were initiated at 24 hours post-challenge and continued for 3 (A) or 7 (B) days or initiated at 38 hours post-challenge and continued for 3 (C) or 7 (D) days. The faint lines are the weights of individual animals recorded throughout the study and the thick lines are the means of the groups ± SEM. The data was analysed by the linear model and the p values shown are the differences between the treatments over time. In addition, Bonferroni's multiple comparisons were used to compare groups at specific time points. * p<0.05 ** p<0.01.

References

1. Hamblin KA, Armstrong SJ, Barnes KB, Davies C, Laws TR, Blanchard JD, Harding SV, Atkins HS. 2017. Inhaled liposomal ciprofloxacin protects against a lethal infection in a murine model of pneumonic plague. Front Microbiol 8, 91.
2. Lettieri JT, Rogge MC, Kaiser L, Echols RM, Heller AH. 1992. Pharmacokinetic profiles of ciprofloxacin after single intravenous and oral doses. Antimicrob Agents Chemother 36, 993–996.
3. Barnes KB, Hamblin KA, Richards MI, Laws TR, Vente A, Atkins HS, Harding SV. 2017. Demonstrating the protective efficacy of the novel fluoroquinolone finafloxacin against an inhalational exposure to *Burkholderia pseudomallei*. Antimicrob Agents and Chemother 61 (7) 1-17.
4. Patel H, Andresen A, Vente A, Heilmann H-D, Stubbings W, Seiberling M, Lopez-Lazaro L, Pokorny R, Labischinski H. 2011. Human Pharmacokinetics and Safety Profile of Finafloxacin, a New Fluoroquinolone Antibiotic, in Healthy Volunteers. Antimicrob. Agents Chemother 55 (9) 4386–4393.
